# Neuroanatomical substrates of action perception and understanding: an anatomic likelihood estimation meta-analysis of lesion-symptom mapping studies in brain injured patients

**DOI:** 10.3389/fnhum.2014.00344

**Published:** 2014-05-30

**Authors:** Cosimo Urgesi, Matteo Candidi, Alessio Avenanti

**Affiliations:** ^1^Laboratorio di Neuroscienze Cognitive, Dipartimento di Scienze Umane, Università di UdineUdine, Italy; ^2^Istituto di Ricovero e Cura a Carattere Scientifico “Eugenio Medea,” Polo Friuli Venezia Giulia, San Vito al TagliamentoPordenone, Italy; ^3^Dipartimento di Psicologia, Università “Sapienza” di RomaRome, Italy; ^4^IRCCS Fondazione Santa LuciaRome, Italy; ^5^Dipartimento di Psicologia e Centro studi e ricerche in Neuroscienze Cognitive, Alma Mater Studiorum - Università di BolognaCampus di Cesena, Italy

**Keywords:** action perception, action simulation, action understanding, mirror neurons, brain lesion, voxel-lesion-symptom mapping, activation likelihood estimation (ALE) meta-analysis

## Abstract

Several neurophysiologic and neuroimaging studies suggested that motor and perceptual systems are tightly linked along a continuum rather than providing segregated mechanisms supporting different functions. Using correlational approaches, these studies demonstrated that action observation activates not only visual but also motor brain regions. On the other hand, brain stimulation and brain lesion evidence allows tackling the critical question of whether our action representations are necessary to perceive and understand others’ actions. In particular, recent neuropsychological studies have shown that patients with temporal, parietal, and frontal lesions exhibit a number of possible deficits in the visual perception and the understanding of others’ actions. The specific anatomical substrates of such neuropsychological deficits however, are still a matter of debate. Here we review the existing literature on this issue and perform an anatomic likelihood estimation meta-analysis of studies using lesion-symptom mapping methods on the causal relation between brain lesions and non-linguistic action perception and understanding deficits. The meta-analysis encompassed data from 361 patients tested in 11 studies and identified regions in the inferior frontal cortex, the inferior parietal cortex and the middle/superior temporal cortex, whose damage is consistently associated with poor performance in action perception and understanding tasks across studies. Interestingly, these areas correspond to the three nodes of the action observation network that are strongly activated in response to visual action perception in neuroimaging research and that have been targeted in previous brain stimulation studies. Thus, brain lesion mapping research provides converging causal evidence that premotor, parietal and temporal regions play a crucial role in action recognition and understanding.

## INTRODUCTION

Ever since the revolutionary proposal that action and perception systems are tightly linked along a continuum rather than being segregated mechanisms supporting different functions, behavioral studies have shown the many ways in which activity in the motor system modulates concurrent or delayed action perception and the other way around (Prinz, 1997; Schütz-Bosbach and Prinz, 2007a). The original idea that action observation triggers a corresponding activation of similar movement in a passive observer dates back to the ideomotor theories developed by Lotze (1852) and James (1890). More recently, a number of behavioral studies have described “compatibility” (facilitatory) and “incompatibility” (inhibitory) effects between an observed movement or posture and an executed movement (see Hommel, 2010 and Heyes, 2011 for reviews), suggesting a bidirectional influence of action observation on motor performance and of action execution on action perception.

### NEURAL CORRELATES OF ACTION PERCEPTION

The actions of others represent a dynamic and extremely complex visual stimulus and posit a strong challenge to the brain for their perception and understanding. In line with the old ideomotor principle, current models of action perception suggest that in order to solve this computational challenge the brain has evolved an efficient sensorimotor mechanism, namely mapping visual representations of the observed actions onto corresponding motor representations (Rizzolatti and Craighero, 2004; Wilson and Knoblich, 2005; Kilner et al., 2007; Schütz-Bosbach and Prinz, 2007b; Gazzola and Keysers, 2009; Friston et al., 2011; Press et al., 2011; Schippers and Keysers, 2011; Avenanti et al., 2013b; Pezzulo et al., 2013). The activation of motor schemata while observing similar motor schemata in others may allow an understanding of others’ actions “from inside” (Rizzolatti and Sinigaglia, 2010) and this motor coding of observed actions may be used to predict incoming visual signals and refine visual perception.

Attention to the action observation–execution coupling gained strong momentum when a plausible neural underpinning of such mechanism was first described under the form of neurons in the F5 sector of the ventral premotor cortex of awake monkeys (di Pellegrino et al., 1992). These cells have been termed “mirror neurons” (Gallese et al., 1996) for their capability to online mirror (i.e., replicate) in motor terms the observed hand–mouth actions (see Casile, 2013 for a review of 20 years of research on mirror neurons in the monkey brain). At their first description in monkeys, the activity of these cells seemed to be strictly dependent upon the actions having a clear transitive goal (i.e., grasping a piece of food), although premotor mirror neurons coding communicative mouth gestures (e.g., lipsmaking; Ferrari et al., 2003) or intransitive hand movements (Kraskov et al., 2009) have been also described. More recently, neurons coding the end-goal of a chain of actions have been described in the inferior parietal cortex of monkeys (i.e., in the cytoarchitectonic area PF and PFG) observing grasp-to-place and grasp-to-eat actions (Fogassi et al., 2005). An important feature of these cells is that their activity seems not to be strictly linked to the precise time-deployment of the observed action; indeed, a certain proportion of parietal mirror neurons are activated in advance of achievement of the end-goal, e.g., during the initial grasping phase (Fogassi et al., 2005). This anticipatory feature was also shown in a single-cell study where monkey premotor mirror neurons fired both when directly seeing hand–food contact and when merely inferring that the observed hand was going to grasp a piece of food behind an occluder (Umiltà et al., 2001).

Transcranial magnetic stimulation (TMS) studies assessing corticospinal excitability (Fadiga et al., 1995; Urgesi et al., 2006, 2010; Candidi et al., 2010; Borgomaneri et al., 2012, 2013, 2014; Barchiesi and Cattaneo, 2013; Mattiassi et al., 2014), electro- and magneto-encephalography (Hari et al., 1998; Cochin et al., 1999; Järveläinen et al., 2004; van Schie et al., 2004; Pineda, 2005; Kessler et al., 2006; Bufalari et al., 2007), functional brain imaging (Chong et al., 2008; Etzel et al., 2008; Kilner et al., 2009; Caspers et al., 2010; Oosterhof et al., 2010; Arnstein et al., 2011; Molenberghs et al., 2012a; Azevedo et al., 2013) and single-cell recording studies in humans (Mukamel et al., 2010) suggested the presence of fronto–parietal neural networks supporting similar mirror-like mechanisms.

A supposed cortical pathway for observed actions to be translated in their motor counterpart (i.e., the action observation–execution link) involves an early processing in visual regions, including the superior temporal sulcus (STS) and the surrounding middle/superior temporal gyri. Monkey studies indicate the STS region contains neurons that are activated by the observation of complex motion conveyed by biological entities (i.e., biological motion) even in the absence of a direct view of the form of the agent that performs the action (Puce and Perrett, 2003). The proposed idea is that visual information coming from lower-level visual areas is sent to temporal regions from where it is relayed to parietal regions (including the inferior parietal lobe and the anterior intraparietal area) and ultimately to premotor regions (Nishitani et al., 2004; Rizzolatti and Craighero, 2004; Caspers et al., 2010; Nelissen et al., 2011; Keysers and Gazzola, 2014). Recent work in humans also suggest that the somatosensory cortex participates in this network (Gazzola and Keysers, 2009; Caspers et al., 2010; Jacquet and Avenanti, 2013; Keysers et al., 2010); however, the pathway through which this region would receive visual signals conveying action observation has been less directly explored.

This temporal, parietal and premotor network, which is often referred to as the action observation network (AON), is suggested to be the basis for sophisticated cognitive skills such as the ability to perceive and understand others’ actions and intentions. Neurophysiological and brain imaging techniques have been essential in highlighting that action observation triggers activation of not only temporal, but also fronto–parietal areas possibly coding visual representation of the observed action in motor terms. However, the correlational approach of these methods cannot establish whether neural activity in the AON is also necessary for action perception and understanding. Thus, to test the causal role of the AON in action perception is fundamental to resort to causal methods, i.e., by investigating the influence of altered neural activity in key nodes of the AON, introduced by brain lesions or non-invasive brain stimulation, on the ability to recognize and understand the actions of others (Avenanti and Urgesi, 2011; Urgesi and Avenanti, 2011; Avenanti et al., 2013b).

### BRAIN STIMULATION STUDIES OF ACTION PERCEPTION

Based on the idea that the activation of motor regions is not only concomitant to action observation but that it plays a causal role in processing and full understanding of others’ behavior, brain stimulation methods, especially repetitive TMS, have been used to highlight the causative role of premotor and motor regions in the visual perception of seen postures and movements (review in Avenanti et al., 2013b). These studies showed that interferential TMS over the inferior frontal cortex [including the posterior part of the inferior frontal gyrus (IFG) as well as the ventral premotor cortex], but not over control regions, impaired the performance of healthy participants during: (i) *biological motion perception*, in which participants are required to blend the coherent motion pattern of a series of point-lights into a unitary perception of a moving person (van Kemenade et al., 2012); (ii) *visual action discrimination*, in which participants are involved in delayed matching-to-sample of static pictures depicting hand grips (Jacquet and Avenanti, 2013), upper or lower limb actions (Urgesi et al., 2007b; Candidi et al., 2008) or whole body movements (Urgesi et al., 2007a); (iii) *weight estimation*, in which participants are presented with videos of an actor lifting and placing a box of different weights and are asked to estimate the weight of the box (Pobric and Hamilton, 2006); (iv) *goal recognition*, in which participants are required to match the end-goal of action videos (Jacquet and Avenanti, 2013); (v) *deception detection*, in which participants are required to recognize whether the actor who lifts an object is trying to provide deceiving information about its weight (Tidoni et al., 2013). Furthermore, repetitive TMS of the inferior frontal cortex during the observation of others’ hand actions prevented healthy participants to perform proactive eye movements similar to those made by the model performing such actions (Costantini et al., 2014; see also Elsner et al., 2013). In a similar vein, stimulation of the inferior frontal cortex abolished the facilitation of motor excitability during action observation (as evidenced by perturb-and-measure TMS protocols: Avenanti et al., 2007, 2013a) as well as the effect of repeated action execution on categorization of seen actions (as shown by cross-modal TMS adaptation; Cattaneo et al., 2011).

Clearly, the functions addressed by these studies are very disparate and involve different levels of action representations, from pure visual processing (e.g., biological perception; discrimination of static postures), active simulation of actor’s efforts in lifting the object (e.g., weight estimation), anticipatory coding of what the actor is doing (e.g., proactive gaze), inference of the action goals independently of their means (e.g., goal recognition) or of the ultimate actor’s intention (e.g., deception detection). It is, thus, unclear at which level and for which specific function does the inferior frontal cortex play a critical role. Furthermore, other studies have shown that action perception and goal recognition are affected not only by stimulation of the inferior frontal cortex, but also by stimulation of the anterior intraparietal cortex (Cattaneo et al., 2010) and of the dorsal premotor cortex (Stadler et al., 2012; Makris and Urgesi, 2014). Similarly, dual coil TMS paradigms show that stimulation of parietal (Koch et al., 2010) and dorsal premotor (Catmur et al., 2011) cortices influences motor excitability during action observation, in a way that is similar to that caused by stimulation of the inferior frontal cortex (Koch et al., 2010; Catmur et al., 2011). Finally, it is also worth noting that performance in some action perception tasks is impaired after stimulation of the temporal nodes of the AON; for example, repetitive stimulation of STS reduces the sensitivity of biological motion perception (Grossman et al., 2005; van Kemenade et al., 2012), alters the ability to detect small postural changes in neutral and angry body images (Candidi et al., 2011), and disrupts the recognition of the outcome of complex sport actions (Makris and Urgesi, 2014). On the other hand, tasks involving the representation of abstract action goals independently of the effector are affected by stimulation of fronto–parietal but not of temporal areas (Cattaneo et al., 2010). Overall, these findings suggest that action perception and understanding rely on different regions which might provide complimentary contributions to the observer’s action representation along a continuum from processing of kinematic features of the observed movement to processing of action goal and intention.

The crucial role played by each node of the AON in action representation, however, cannot be fully clarified by brain stimulation studies alone since the interference induced by single dose TMS of a given area might determine transient functional fluctuations of networks’ activity (Siebner et al., 2009; Avenanti et al., 2012a,b; Arfeller et al., 2013). It is likely that such transient instabilities trigger fast compensatory functional reorganization of the network (Arfeller et al., 2013; Avenanti et al., 2013a), as documented for other domains such as action selection (O’Shea et al., 2007), thus allowing task performance to recover (Sack and Linden, 2003; Siebner et al., 2009; Reithler et al., 2011). These patterns of results would somehow limit the implication of brain stimulation results to the description of action perception and understanding deficits in chronic clinical conditions, associated to either neurodevelopmental disorders (e.g., autism spectrum disorder) or acquired brain damage (e.g., apraxia). Indeed, although plastic mechanisms are also evident after these latter forms of lesions, it is clear that these changes are completely different in both their nature and timing and imply extremely different functional effects from those consequent to brain stimulation methods. For example, while real lesions generally induce both morphological and functional long-term changes, virtual lesions induce faster functional changes that vanish away within the time of milliseconds to minutes at the most.

Thus, to establish the causal role of key nodes of the AON in action perception it is fundamental to provide convergent evidence from brain stimulation and brain lesion methods. In addition, although non-invasive brain stimulation techniques allow studying the effects of transient alterations of activity in motor cortical areas on their visual perception, one important limit of this method is that it cannot be applied to deep brain regions as only superficial areas can be easily stimulated. Critically, thus, brain lesions are the only way to describe any stable and causal role of superficial and non-superficial AON areas to action perception and understanding. Overall, the description of the neuropsychological deficits in brain lesion patients provides information on the functions that cannot be, or are much more difficult to, recover after damage to a given gray or white matter area. This provides more compelling evidence for the comprehension of the neural bases of action perception and understanding.

### PIONEER NEUROPSYCHOLOGICAL STUDIES ON ACTION PERCEPTION DEFICITS

The investigation of action perception and understanding disorders in brain lesion patients started from the pioneering findings of two classical research streams documenting action perception disorders in patients suffering from aphasia and apraxia, respectively.

The notion that patients with aphasia present disturbances also in pantomime recognition dates back to the seminal clinical observations of Finkelnburg (1870; cited in Varney, 1978), Jackson (1878; cited in Varney, 1978), and Head (1926; cited in Varney, 1978) and was attributed to a general deficit in symbolic thinking (asymbolia). Further studies, however, provided contrasting evidence that pantomime recognition deficits in aphasia patients correlate with the severity of their linguistic deficits. Duffy and Duffy (1975, 1981) developed a pantomime recognition test that did not require processing of verbal instructions or production of a verbal response and patients had simply to point to the correct gesture; they found that patients with aphasia were more impaired than patients with right hemisphere (RH) or subcortical damage and their pantomime recognition abilities correlated with their overall linguistic competence. On the other hand, some studies showed that pantomime recognition in aphasics was independent from general linguistic deficits (Gainotti and Lemmo, 1976) and was more associated to deficits in reading than to deficits in oral comprehension, suggesting a link of pantomime recognition deficits with visual rather than linguistic or “symbolic” processing (Varney, 1978, 1982). Furthermore, qualitative analysis of the errors made by the aphasic patients in pantomime recognition demonstrated that they most often selected the semantic distractor, suggesting a specific difficulty in extracting the correct meaning of pantomimes (Varney and Benton, 1982; Duffy and Watkins, 1984). Finally, preliminary attempts to identify the neural correlates of pantomime recognition deficits in aphasia (Varney and Damasio, 1987; Varney et al., 1989) revealed that they resulted from lesions in basal ganglia and posterior temporo–parietal cortices, although the association between lesion of these areas and pantomime recognition deficits was weak (i.e., many patients with lesions in these areas did not exhibit any deficit).

The second research stream on the links between motor dysfunctions and action perception-understanding deficits originated the finding that patients with limb apraxia have deficits not only in imitating observed gestures, but also in distinguishing between well-performed from poorly performed movements (Heilman et al., 1982) and in understanding their meaning (Rothi et al., 1985). Importantly, action perception and understanding disorders were specific to the apraxia patients with posterior lesions, while those with anterior lesions were unaffected. In a similar vein, patients with ideational apraxia (defined as the inability to demonstrate correct object-use), presented deficits in sequencing pictures of object-use actions but not of other common events not requiring object manipulation; the deficits in action sequencing were independent from the severity of aphasia or ideomotor apraxia (i.e., gesture imitation) deficits (Lehmkuhl and Poeck, 1981; see also Rapcsak et al., 1995). These findings were interpreted in the context of a dissociation between conceptual action disturbances, which follow left parietal lesions and reflect the disruption of “visuo-kinesthetic motor engrams” guiding the sequencing and timing of motor movements, and production deficits, which follow premotor lesions and reflect the disconnection between parietal centers and motor production system (Heilman et al., 1997; Goldenberg, 1999; Stamenova et al., 2012). Following the same research stream, however, Halsband et al. (2001) found that patients with lesions involving the left parietal cortex showed severe action production and imitation impairments, but only slight, if any, deficits in tasks requiring to judge whether a given sequence was correctly or inadequately performed, to detect sequence or performance errors, or to identify the missing link in an incomplete sequence; conversely, patients with left premotor lesions or RH lesions were not affected in either action comprehension or production.

Overall, classical neuropsychological studies provided evidence that action comprehension disorders may be associated to language or imitation deficits in left hemisphere (LH) patients with aphasia and/or apraxia. All these studies highlighted a certain degree of variability among aphasia and apraxia patients in their relative performance in action comprehension tasks, suggesting that different brain lesions may induce associated or dissociated patterns of action comprehension and production disorders. The scanty documentation about lesion extent and localization notably limited the anatomical inferences that could be drawn from these findings. Recent neuropsychological studies have strengthened the investigation of the neuroanatomical correlates of action perception and understanding disorders by using lesion mapping and analysis methods that allow testing the extent of the association between lesions in a given brain region and specific behavioral deficits. Performing a systematic review of these studies in order to identify pattern of consistent associations between specific brain lesions and action perception and understanding disorders is the aim of the present study.

### THE PRESENT STUDY

In the present study, we aimed to perform an anatomic likelihood estimation (AnLE) meta-analysis of studies using formal lesion-symptom mapping methods to describe the causal relation between brain lesions and action perception and understanding deficits. We considered studies using any formal lesion-symptom mapping procedures spanning from statistical frequency comparison of the lesion overlaps of impaired vs. non-impaired patients (Rorden and Karnath, 2004) to voxel-lesion-symptom mapping (VLSM) according to which, for each brain voxel, the performance of damaged patients is compared to that of non-damaged patients (Bates et al., 2003; Rorden et al., 2009), and comprising also voxel-based morphometry (VBM), which correlates gray-matter density to behavioral performance (Ashburner and Friston, 2000). The quantitative approach of these methods allows investigating subtle and continuous action perception and understanding deficits and associating them with their specific neural substrate.

A limitation of lesion mapping analyses of single studies is that their results are strictly dependent not only on the behavioral task used to probe action perception and understanding skills, but also on the patient population entered into the analysis. In fact, previous studies used different sets of tasks, which relied to different extent on motor production, visual perception and language processing, thus making it difficult to compare the results and to exclude the contribution of deficits attributable to damage to primary sensorimotor areas and/or language areas. Furthermore, the neuroanatomical inferences that can be drawn from the results of these single studies are stronger as more patients with disparate lesion localization and extent are entered into the analysis. However, having a high number of patients satisfying the inclusion criteria for reliable neuropsychological evaluation and with acceptable neuroradiological lesion documentation is one of the major issues in neuropsychological research. As a reflection of this issue, previous studies focused on subpopulations of patients selected on the basis of a specific symptom (e.g., apraxia or aphasia) or on the basis of lesion localization (left or right hemisphere). Since the number of patients in the different studies is relatively small and not surely optimal to cover all brain areas with acceptable power, we believe that formal meta-analytic works may facilitate the emergence of a consistent pattern of association between specific brain lesions and action perception and understanding disorders.

We thus performed a systematic review of existing studies investigating the neuroanatomical substrate of action perception and understanding disorders in brain lesion patients and used BrainMap Ginger ALE 2.3 software (http://brainmap.org) to perform an AnLE meta-analysis. Although Ginger ALE was developed for activation likelihood estimation (ALE) meta-analyses when used in conjunction with functional neuroimaging results (Turkeltaub et al., 2002; Laird et al., 2005), it also allows performing AnLE meta-analyses if used in conjunction with anatomic data such as VBM (e.g., Nickl-Jockschat et al., 2012) or VLSM (e.g., Chechlacz et al., 2012; Molenberghs et al., 2012b). This last method assesses the overlap between anatomical foci identified by different research groups using voxel-wise analyses of the foci obtained based on various lesion-symptom mapping approaches. In the present context, the results of the meta-analysis allowed identifying consistent associations between brain damage and action perception and understanding deficits.

## MATERIALS AND METHODS

### LITERATURE SEARCH AND SELECTION CRITERIA

For the purpose of the present study we performed a systematic search in the literature to identify all the relevant papers reporting the performance of brain lesion patients in action perception and understanding tasks. To avoid over-selecting the list on the basis of the specific lesion analysis used, an initial search identified all studies published after 2001 and investigating action perception in brain lesion patients. We searched PubMed with the following keywords: [(action OR actions OR gestures OR gesture OR pantomime OR pantomimes OR “biological motion”) AND (perception OR discrimination OR prediction OR understanding OR recognition OR knowledge OR comprehension OR observation OR recognition) AND (“brain lesion” OR “brain damage” OR “brain injury” OR “brain lesioned” OR “brain damaged” OR “brain injured” OR “hemisphere lesion” OR “hemisphere damage” OR “hemisphere injury” OR “hemisphere lesioned” OR “hemisphere damaged” OR “hemisphere injured” OR “brain stroke” OR “hemisphere stroke” OR aphasia OR apraxia OR agnosia) AND (publication date > 2001) NOT (review)]. This yielded a list of 415 papers (last update 11 December 2013), which were screened to select the papers satisfying the following inclusion criteria: (1) testing the performance of focal brain lesion patients (e.g., studies on degenerative or neurodevelopmental disorders were not included); and (2) using at least one action perception and/or understanding task. We identified 34 original research articles published after 2001 that tested action perception in focal brain injured patients and administered at least one action perception and/or understanding task. The reference list of these papers was screened to identify other papers not picked up by the previous automatic search. This allowed us to identify other two papers (Battelli et al., 2003; Tranel et al., 2003). The resulting list of 36 papers was then screened for the following exclusion criteria: (1) not mapping and analyzing patients lesions using one of the standard lesion-symptom mapping approaches based on VLSM, subtraction of lesion overlaps, or VBM; (2) administering tasks with strong linguistic processing demand (e.g., action naming or verb to action scene matching) and (3) cases in which the coordinates of the clusters in the Montreal Neurological Institute (MNI; Evans et al., 1993) or Talairach space (Talairach and Tournoux, 1988) could not be identified either from the information provided in the paper or directly from the authors. Based on these exclusion criteria we did not include studies that involved only single case analyses or a few patients and that selected the patient group on the basis of the presence of a specific symptom associated to the experimental task (i.e., studies where no statistical comparison with a different patient group was performed).

Twelve papers (Sörös et al., 2003; Yoon et al., 2005; Arévalo et al., 2007, 2011, 2012; Bi et al., 2007; Negri et al., 2007; Tranel et al., 2008; Papeo et al., 2010; Pillon and d’Honincthun, 2011; Vannuscorps and Pillon, 2011; Stamenova et al., 2012) were not considered because their action understanding tasks required processing of linguistic stimuli, either naming of visually presented actions or word to picture matching that involved understanding of the word meaning. Five papers were not considered further because they reported single case analyses of action perception and understanding disorders in patients with agnosia (Huberle et al., 2012; Moro et al., 2012), apraxia (Sunderland, 2007), aphasia (Cocks et al., 2009), or frontal brain lesion (Eskenazi et al., 2009). Three papers were not included because they studied small groups of patients who were all impaired in biological motion detection (three patients in Battelli et al., 2003), in sequencing observed actions (six patients in Fazio et al., 2009) or in matching mouth action sounds (Schmid and Ziegler, 2006) and no VLSM or lesion subtraction statistical analysis could be performed. Two studies (Serino et al., 2010; van Dokkum et al., 2012) could not be included because no lesion mapping was performed and patients were recruited on the basis of specific motor symptoms (hemiplegia) whose presence was associated to performance in the experimental task (perception of biological motion). Finally, three studies (Tranel et al., 2003; Kemmerer et al., 2012; Rogalsky et al., 2013) were not included in the meta-analysis because the coordinates of the foci associated to action perception and understanding deficits were not available. From the list of 36 papers published after 2001 and testing action perception and recognition in brain lesion patients, we thus identified 11 papers that did not meet any exclusion criteria (see **Table 1**).

**Table 1:** List of studies considered for the AnLE meta-analysis listed in chronological order.

No	Study	Patients	Damaged hemisphere	Major disorder	Task	Analysis	Statistics	Foci provided
1	Saygin et al. (2004a)	29	LH	Aphasia	Visuo-visual matching of actions	VLSM	*t*-test	3 (IFC, S1, caudate)
2	Buxbaum et al. (2005)	24	LH	Apraxia	Detection of action spatial errors	Subtraction	χ^2^	2 (IPC)
3	Saygin (2007)	47*	LH	Mixed deficits	Detection of biological motion (whole body)	VLSM	*t*-test	2 (IFC, MTC/STC)
4	Moro et al. (2008)	28	14 LH, 11 RH, 3 Bil	Mixed deficits	Visuo-visual matching of action pictures (upper and lower limbs)	VLSM	BM	2 (L-IFC, R-IFC)
5	Pazzaglia et al. (2008a)	28	LH	21 apraxia	Sound-to-picture matching of actions (upper limbs and mouth)	VLSM	BM	6 (4 IFC, 2 IPC)
6	Pazzaglia et al. (2008b)	33*	LH	21 apraxia	Error detection in action videos	Subtraction and VLSM	χ^2^ and BM	3 (IFC)
7	Weiss et al. (2008)	16*	LH	9 apraxia	Detection of sequencing error in action videos	Subtraction	χ^2^	1 (IPC)
8	Kalénine et al. (2010)	43	LH	Mixed deficits	Detection of spatial action errors	VLSM	*t*-test	4 (2 MFC, IPC, MTC/STC)
9	Nelissen et al. (2010)	13	LH	13 aphasia	Error detection in action videos	VBM	Regression	1 (MTC/STC)
10	Han et al. (2013)	77	26 LH, 15 RH, 36 Bil	Mixed deficits	Matching of point-light animations with action pictures (whole body)	VLSM	BM	2 (MFC, MTC/STC)
11	Kalénine et al. (2013)	23	LH	17 apraxia	Discrimination of means differences between two action videos	VLSM	*t*-test	4 (2 IPC, 2 MTC/STC)

*
Bil, bilateral; BM, Brunner–Munzell; IFC, inferior frontal cortex (including posterior inferior frontal gyrus and ventral premotor cortex); IPC, inferior parietal cortex (including supramarginal and angular giri and intraparietal sulcus); LH, left hemisphere; MFC, middle frontal cortex (including the middle frontal gyrus and the dorsal aspect of the precentral gyrus, e.g., dorsal premotor and motor cortices); MTC/STC, middle and superior temporal cortex; RH, right hemisphere; S1, primary somatosensory cortex; VLSM, voxel-lesion-symptom mapping. Asterisk indicates that the lesion analysis in the original study was performed only on a subset of patients and the number of patients considered here is that entered into the analysis and not of the study whole sample. Except when otherwise specified, upper limb actions were used as stimuli.*

### DATA ANALYSIS

Based on the results of the literature search we entered all the foci whose coordinates (1) were reported by the authors in the paper, (2) could be identified from the information provided in the paper, or (3) were provided by the authors as personal communication. The center coordinates of all clusters reported in the papers were considered provided they referred to tasks involving action perception and understanding independent of linguistic coding. Thus, the coordinates of clusters associated to all tasks were included in cases in which multiple action perception tasks were administered to patients. Conversely, the coordinates of foci associated to tasks requiring linguistic coding (e.g., picture to word matching as in the semantic task in Buxbaum et al., 2005 and Kalénine et al., 2010) were not included in the analysis to rule out the spurious lesional effects of areas associated to language disorders. In cases in which multiple analyses were performed on the same data set but using different lesion analysis approaches (e.g., Pazzaglia et al., 2008b), we entered the coordinates resulting from all analyses. For each cluster, the coordinates of the voxel with maximal statistical value or of the center of mass were entered into the analysis, according to which of the two coordinates was provided by the authors.

We performed all analyses in MNI space and the coordinates originally reported in Talairach space were converted into MNI space with the coordinate conversion tool implemented in Ginger ALE software which uses the best-fit icbm2tal transform (Lancaster et al., 2007). We used the revised version of the AnLE methods (Eickhoff et al., 2009) which considers random effects and incorporates variable uncertainty based on sample size. Furthermore, a modification to the AnLE method (Turkeltaub et al., 2012) was used to limit the effect of a single experiment and minimize within-group effects. In keeping with previous AnLE meta-analyses on brain lesion mapping data (e.g., Chechlacz et al., 2012; Molenberghs et al., 2012b), this modified AnLE algorithm was used to control for dependent within-group effects in studies providing different sets of coordinates based on different data analysis approaches (e.g., lesion overlap subtraction and VLSM; as in Pazzaglia et al., 2008b) or on different action perception tasks administered to the same group of patients (as in Pazzaglia et al., 2008a). This AnLE approach models the anatomical foci from different published reports as Gaussian probability density distribution at a given coordinate and calculates the Modeled Anatomic maps (i.e., the 3D images of each foci group) on the basis of the maximum across each focus’s Gaussian. Then, an experimental AnLE map is created from the voxel-wise union of all Modeled Anatomic maps. Differentiation of true concurrence of foci vs. random spatial association is performed by testing the experimental AnLE map against AnLE null distribution maps that are generated utilizing a permutation test of randomly generated foci. For thresholding purposes, we followed a cluster level inference method (Eickhoff et al., 2012), which sets the cluster minimum volume such that only 5% of the simulated data’s clusters exceed this size. This way, we avoided setting *a priori* a minimum cluster size which could have removed small clusters with high convergence of studies. A cluster-forming statistical threshold of *p* < 0.05 FDR (false discovery rate) was used to correct for multiple comparisons. The resulting maps were overlaid onto the T1-weighted template MRI scan from the MNI provided with the MRIcron software (Rorden and Brett, 2000; available at http://www.mricro.com/mricron). The anatomical localization of the significant clusters identified by the meta-analyses was based on probabilistic cytoarchitectonic maps of the human brain using the SPM Anatomy Toolbox v. 1.7 (Eickhoff et al., 2005). Using a Maximum Probability Map, foci were assigned to the most probable histological area at their respective locations.

## RESULTS

The 11 studies and foci entered into the meta-analysis are reported in **Table 1**. The studies involved a total of 361 patients and reported 30 foci of significant lesion-deficit associations. Most patients had lesions in the LH (*N* = 296); only two studies (Moro et al., 2008; Han et al., 2013) reported and analyzed also patients with RH (*N* = 26) and bilateral posterior (*N* = 39) lesions; two further studies (Saygin, 2007; Weiss et al., 2008) tested both LH and RH patients but did not include RH patients in the lesion mapping analysis. Within the LH group, however, there was a good coverage of frontal, parietal, and temporal lesions.

**FIGURE 1:**
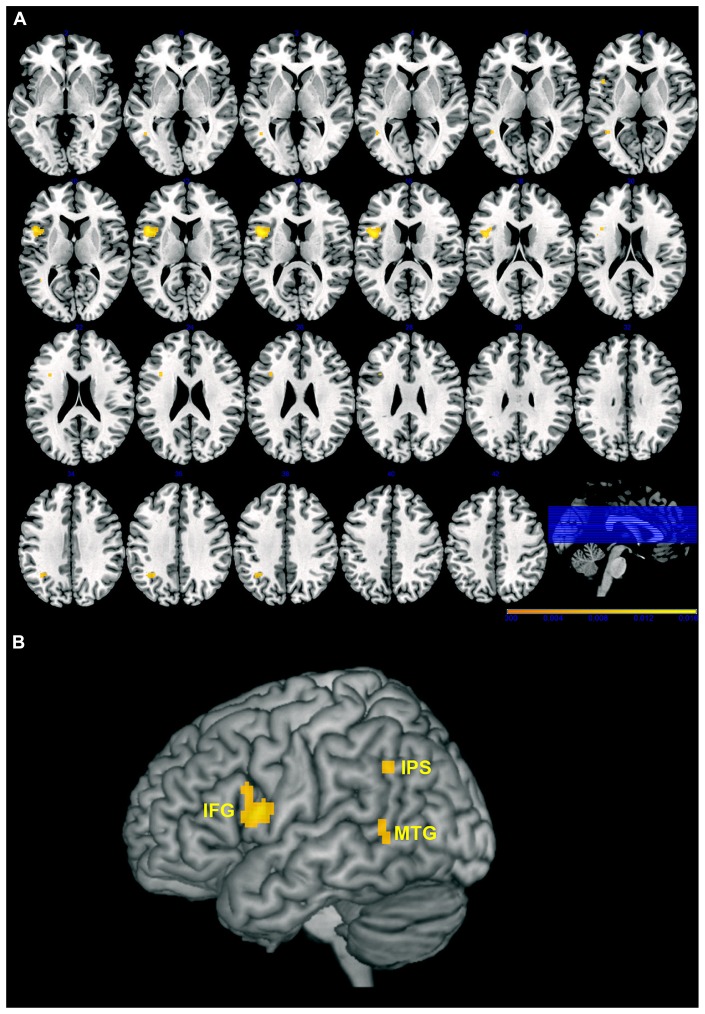
**Maps of the clusters with significant association between brain lesions and action perception, and understanding disorders overlaid on axial slices (A) or 3D rendering (B) of the Montreal Neurological Institute (MNI) template.** Left hemisphere is on the left, and right hemisphere is on the right. Color scale indicates AnLE value range. IFG, inferior frontal gyrus; IPS, intraparietal sulcus; MTG, middle temporal gryus. Note that deeper regions are projected onto the surface of the template to better highlight the extension of the cluster.

The results of the AnLE meta-analysis are listed and detailed in **Table 2** and they are displayed in **Figure 1**. We identified three lesion clusters with significant co-occurrence of associations with action perception and understanding disorders. The largest cluster (1920 voxels) was located in the left frontal cortex (MNI coordinates of the weighted center, *x, y, z*: -44, 10, 14) and was assigned to Brodmann area (BA) 44 (30.4% of the cluster voxels) and BA 45 (3.4% of the cluster voxels). Local maxima were identified in the pars opercularis (MNI: -48, 12, 12) and pars triangularis (MNI: -38, 14, 26) of the IFG and in the rolandic operculum (MNI: -42, 6, 14). The other two clusters were much smaller. One cluster (304 voxels) was located in the left inferior parietal cortex (MNI coordinates of the weighted center, *x, y*, *z*: -35, -54, 36) and was assigned mostly to human intraparietal area 1 (hIP1; 57.2% of the cluster voxel) and marginally to hIP3 (0.7% of the cluster voxels). The third cluster was located in the left middle/superior temporal cortex (MTC/STC) and centered on the lower bank of the STS (MNI coordinates of the weighted center, *x*, *y*, *z*: -43, -52, 5); local maxima were identified in the middle temporal gyrus (MNI: -42, -52, 8) and the underlying white matter (MNI: -44, -52, 2). The cluster with greatest convergence was the one in the IFG (AnLE value = 0.017), especially in the pars opercularis, while the other two clusters were less reliably identified in the studies considered here (AnLE value < 0.12).

**Table 2:** Significant AnLE clusters and MNI coordinates of the corresponding local maxima identified in the inferior frontal cortex (IFC), inferior parietal cortex (IPC), and middle/superior temporal cortex (MTC/STC).

Cluster no	Volume (mm^3^)	Weighted center (MNI *x,y,z*)	Macroanatomical location	Cytoarchitectonic location	AnLE max value	MNI coordinates		
						*x*	*y*	*z*
1	1920	(-44, 10, 14)	Left inferior frontal gyrus	BA 44/BA 45				
			Rolandic opercolum		0.017	-42	6	14
			Inferior frontal gyrus, pars opercolaris		0.017	-48	12	12
			Inferior frontal gyrus, pars triangularis		0.01	-38	14	26

2	304	(-35, -54, 36)	Anterior intraparietal sulcus	hIP1/hIP3				
					0.012	-36	-54	36

3	192	(-43, -52, 5)	Left middle temporal gyrus					
					0.01	-42	-52	8
					0.009	-44	-52	2

*
BA, Brodmann area; hIP1, human intraparietal area 1; hIP3, human intraparietal area 3.*

## DISCUSSION

Previous neurophysiological and brain imaging techniques have been essential in demonstrating that observing others’ actions activates high-order visual areas in the temporal cortex, which are involved in processing biological motion, as well as frontal and parietal somatomotor regions, which are involved in performing the observed actions (Puce and Perrett, 2003; Rizzolatti and Craighero, 2004; Caspers et al., 2010; Grosbras et al., 2012). However, these approaches only provide correlational evidence and cannot establish whether temporal, parietal, and frontal areas are necessary for visual recognition and understanding of others’ actions (Avenanti and Urgesi, 2011; Avenanti et al., 2013b).

Our meta-analysis of brain lesion studies investigating the neural correlates of action perception and understanding disorders using quantitative lesion mapping analyses showed that lesions of three crucial nodes of the AON, namely the inferior frontal cortex, inferior parietal cortex, and MTC/STC, are consistently associated to deficits in perceiving and understanding the actions of other individuals. This converges with neurophysiologic, neuroimaging and brain stimulation studies in showing that the ability to understand others’ behavior recruits a large network of temporal, parietal, and premotor areas that may play complimentary roles in the ultimate action representation.

The probabilistic cytoarchitectonic anatomical localization of the three clusters assigned the inferior frontal cortex cluster mostly to BA 44 and only marginally, in its antero-dorsal aspect, to BA 45. This localization corresponds very much to what reported in the previous ALE meta-analysis of functional imaging studies carried out by Caspers et al. (2010) and it converges with the region we identified in a previous review of the literature of brain stimulation studies that investigated the neural substrates of action perception (Avenanti et al., 2013b). Moreover, the BA44 region is thought to be the human homolog of the macaque ventral premotor cortex area F5 where mirror neurons where first described in the monkey brain (di Pellegrino et al., 1992; Rizzolatti and Craighero, 2004). This convergence provides compelling evidence for a critical role of the inferior frontal cortex in action perception.

The inferior parietal cortex cluster was assigned to hIP1 and marginally to hIP3. Thus our parietal cluster resulted to be located more posteriorly and medially than the rostral inferior parietal area (area PFt), which represented the most anterior part of the parietal cluster identified by Caspers et al. (2010) and might correspond to area PF of the monkey brain (Caspers et al., 2008), where parietal mirror neurons were identified (Fogassi et al., 2005). However, parietal mirror neurons have been reported also more posteriorly, in area PFG (Fogassi et al., 2005; Bonini et al., 2010) and monkey imaging studies show that action observation triggers activity not only in area PF, but also in PFG as well as in the somatosensory and intraparietal cortex (Evangeliou et al., 2009; Nelissen et al., 2011). Remarkably, our hIP1/hIP3 cluster appears to overlap, at least partially, with the most posterior aspects of the parietal cluster identified by Caspers et al. (2010), which, similarly to monkey data, extended to the somatosensory cortex and the intraparietal sulcus (IPS) and more specifically to the cytoarchitectonic area hIP3. This partial convergence between our meta-analysis and previous ALE meta-analysis of functional imaging studies (Caspers et al., 2010) may be due to technical reasons. Indeed, besides issues related to the anatomical resolution of lesion mapping methods, an additional key difference should be considered between neuroimaging and lesion studies. While functional magnetic resonance imaging (fMRI) technique detects activation mainly in the gray matter (at least in its typical applications), lesion studies can reveal behavioral consequences of lesion occurring to both gray and white matter. Considering that our cluster was quite medial (MNI *x* = -36), it is likely that it comprised not only gray matter in the intraparietal cortex but also the underlying white matter and, thus, its connections with other brain regions. Notably, functional and structural connectivity studies suggest that human hIP1 and hIP3 are mostly connected with the inferior frontal cortex (e.g., ventral premotor and IFG; see Uddin et al., 2010), which closely corresponds to our frontal cluster. Thus, these findings would support the notion that inferior fronto–parietal networks support action recognition and understanding.

Finally, regarding the temporal cluster, its location closely corresponded to the cluster in the superior temporal sulcus/posterior middle temporal gyrus that was identified by Caspers et al. (2010), despite being again slightly more medial (i.e., suggesting affection of the white matter underlying the middle temporal gyrus).

An important feature of the present AnLE meta-analysis concerns the inclusion of studies that aimed explicitly to exclude that the action tasks had linguistic demands that could affect performance even if patients with aphasia were tested. Thus, our methodological choice to include only papers administering action perception tasks with low, if any, linguistic processing demands allowed ensuring that language comprehension or production abilities are not confounding our results. As noted for brain stimulation studies, however, brain lesion studies used different types of tasks that demand different levels of action representation, from purely perceptual to goal and intention representation levels. Our AnLE meta-analysis allowed us to detect the clusters more consistently associated to general action perception deficits (independently from any linguistic demands). However, the small number of studies did not allow us to perform a more accurate task analysis to detect specific task-lesion associations and this should be considered a limitation of our study. Nevertheless, we believe that a qualitative description and classification of the tasks used in the different studies reported here may be very helpful in clarifying which functions were tapped on and provide a guide for the functional characterization of the tasks used to study action perception in future studies. In the following, we attempted such a task classification, although it should be kept in mind that our AnLE meta-analysis supports a general involvement of the three clusters in action perception and not their specific functional characterization. Inspection of the tasks used in the different studies suggests that they can be clustered into four different types: (1) biological motion perception (Saygin, 2007; Han et al., 2013); (2) discrimination of action pictures or sounds (Moro et al., 2008; Pazzaglia et al., 2008a; Kalénine et al., 2013); (3) detection of spatio–temporal errors in action sequences (Buxbaum et al., 2005; Pazzaglia et al., 2008b; Weiss et al., 2008; Kalénine et al., 2010; Nelissen et al., 2010); (4) identification of action goal (Saygin et al., 2004a).

### MOVEMENT PERCEPTION

In two studies, perception of biological motion was tested presenting point-light displays of human actions and requiring participants to discriminate them from their scrambled versions (Saygin, 2007) or to associate them to a static picture of the corresponding action (Han et al., 2013). In both studies, the task required the patients to extrapolate human actions from the coherent pattern of motion of dots and both studies found that lesions in the MTC/STC and premotor cortex affected biological motion perception. However, while Han et al. (2013) entered both left and right (and bilateral) lesions into the analysis and found that only RH areas were associated to biological motion perception deficits, Saygin (2007) entered only LH lesions in her quantitative analysis and found a role for both MTC/STC cortex and inferior frontal cortex in the LH. Importantly, the behavioral analysis of RH damaged patients revealed that their performance was also impaired and was comparable to that of LH damaged patients, suggesting no specific lateralization effects in this task. It is possible that the choice of Han et al. (2013) to partial out the word-to-picture matching abilities of patients from the biological motion perception predictor ensured to exclude any effects of linguistic confounds, but may have also masked the deficits shown by LH damaged patients in biological motion perception with respect to RH damaged patients. Overall, the data of both studies are in keeping with neuroimaging evidence that observation of point-light displays of human actions activates not only middle/superior temporal (Grossman et al., 2000; Puce and Perrett, 2003) but also premotor areas (Saygin et al., 2004b) and with brain stimulation evidence that interference with both middle/superior temporal (Grossman et al., 2005) and premotor areas (van Kemenade et al., 2012) disrupts biological motion perception.

While our meta-analysis suggests that both temporal and premotor cortices are critical in perceiving the actions of others, studies suggest these regions may provide complimentary contributes to the extrapolation of human movement information from point-light displays. Single-cell recording shows that neurons in STS and premotor areas have different response properties. Indeed, while both types of cells respond during action observation, no study has so far reported STS neurons responding to both observed and executed actions similar to what mirror neurons in the premotor and parietal areas do (Keysers and Perrett, 2004; Rizzolatti and Craighero, 2004). Rather, some STS neurons appear to decrease their activity during action execution (Keysers and Perrett, 2004). On the other hand, while both STS (Baker et al., 2001) and premotor (Umiltà et al., 2001) neurons continue responding during occlusion of the action, they show a differential pattern of temporal coupling with the action course. Indeed, STS neurons respond to the articulated static postures that correspond to the end-point of the actions but not to their start-point (Jellema and Perrett, 2003a); furthermore, the response of some STS neurons to static body postures is influenced by which action has been previously observed (Jellema and Perrett, 2003b) suggesting that their firing is influenced by the perceptual history of the action sequence in which a body posture is presented (Perrett et al., 2009). Conversely, mirror neurons in the premotor cortex show a more variegate response pattern, with some being activated in advance of goal achievement (Umiltà et al., 2001), others that stop firing when the target object has been reached and grasped, and others continuing to discharge also during the active holding phase (Gallese et al., 1996). Taken together, these results may suggest that, while neural activity in STS and the surrounding MTC/STC uses visual information and perceptual experience to form a representation of ongoing actions (Perrett et al., 2009), activity in the premotor cortex may allow using previous motor experience with similar actions in order to simulate missing or ambiguous visual information on ongoing actions (Wilson and Knoblich, 2005; Urgesi et al., 2012; Avenanti et al., 2013a). This would suggest that the less rich is visual processing in STS the more motor simulation processing in premotor cortex is required to construct a full action representation from ambiguous visual information. Direct evidence for this compensatory plasticity of visual and motor action representation came from a “perturb and measure” TMS study (Avenanti et al., 2013a) showing that motor facilitation during posture observation increases after interferential stimulation of STS (see also Arfeller et al., 2013 for converging TMS-fMRI evidence).

### ACTION DISCRIMINATION

The second group of studies used tasks that require matching two similar static pictures (Moro et al., 2008) or videos (means detection task in Kalénine et al., 2013) of body actions or matching an action sound to its corresponding action picture (Pazzaglia et al., 2008a). The results of these three studies were somehow discrepant, likely depending on the type of actions stimuli used (i.e., transitive vs. intransitive). Indeed, while Moro et al. (2008) used only intransitive or mimicked actions, Kalénine et al. (2013) used only transitive actions and Pazzaglia et al. (2008a) used both transitive and intransitive actions of upper limbs and mouth. In keeping with the brain stimulation studies using a similar task in healthy individuals (Urgesi et al., 2007b; Candidi et al., 2008), Moro et al. (2008) showed that damage to left or right inferior frontal cortex impaired the ability to discriminate two body part pictures on the basis of the specific intransitive action the model was performing. On the other hand, the means difference detection task used by Kalénine et al., 2013 required the comparison of the body movements of two goal-directed transitive actions having similar outcome (e.g., cleaning with a straight or circular movement) and performance in this task was associated to damage to the inferior parietal cortex but not to the inferior frontal cortex. Finally, Pazzaglia et al. (2008a) found that lesions of both inferior frontal and inferior parietal cortex impaired the ability to associate sounds to their corresponding action picture. Overall, these studies appear in keeping with the differential involvement of inferior frontal and inferior parietal cortices in mapping intransitive and transitive actions (Buccino et al., 2001), with the inferior frontal cortex being involved in the encoding of both types of actions and the parietal cortex being more involved in the encoding of goal-directed actions (see also Grafton and Hamilton, 2007; Lestou et al., 2008; Jacquet and Avenanti, 2013).

### ERROR DETECTION

The third group of studies required participants to detect errors in videos of body actions. In two of these studies, patients with apraxia (Pazzaglia et al., 2008b) and aphasia (Nelissen et al., 2010) were required to observe videos of transitive and intransitive actions that could be executed correctly or not. Although the stimuli used in the two studies were the same, Pazzaglia et al. (2008b) used an intermingled presentation of correct and incorrect actions and participants were required to decide whether each action was executed correctly or not; beyond tapping executive functions required to take a decision (see also Kalénine et al., 2013), this task requires matching the observed action to an internal representation of how that action is normally executed, thus likely calling for motor simulation. These specific task requirements were indeed associated to damage to the inferior frontal cortex. Conversely, Nelissen et al. (2010) presented three versions of the same action (two erroneous versions and one correct) and participants were required to decide which of the three versions was correctly executed; the visual presentation of correct and erroneous executions might have facilitated the identification of the correct solution without the need to represent with simulation processes how that action should be executed. Indeed, the authors did not find any association between performance in the task and inferior frontal cortex lesion; on the other hand, performance deficits were associated to damage of the left STC, possibly reflecting the use of visual action processing to solve the task.

In the other three studies of this group (Buxbaum et al., 2005; Weiss et al., 2008; Kalénine et al., 2010), participants were presented with a linguistic description of a transitive action and then observed action videos that could or could not contain errors. While in the spatial task of Buxbaum et al. (2005) and Kalénine et al. (2010) participants had to choose the correctly executed action between two action videos that contained or not spatial errors (spatial task), Weiss et al. (2008) required participants to decide whether each video depicted correctly executed action or actions with spatial or sequencing errors. In both cases, patients’ performance was associated to damage of the inferior parietal cortex/angular gyrus, suggesting a crucial role of this area in representing the correct spatio–temporal profile of transitive actions. Crucially, while both these tasks contained a linguistic cue (the initial description of the action verb or sentence), processing of the linguistic stimuli was almost irrelevant to task performance, since deciding which action contains a spatial or sequencing error is independent from the processing of its linguistic description. On the other hand, we decided to exclude from the Buxbaum et al. (2005) and Kalénine et al.’s (2010) papers the so called semantic task, that required to associate a verb to one of two different correctly executed action videos. Since this task was strictly related to the understanding of the verb meaning, it did not satisfy the exclusion criteria of not being related to linguistic processing. Conversely, the spatial task could be performed also without understanding of the verb meaning.

### ACTION GOALS

The study of the fourth group (Saygin et al., 2004a) required matching the correct objet to a schematic drawing of action. This task does not require the discrimination of the correct action kinematics, but the access to the immediate-goal of observed transitive actions. Performance in this task showed a specific association with damage of the left inferior frontal cortex in aphasia patients, suggesting a role of this area in representing the congruence of action means and goal. It is worth noting that also the so-called outcome detection task in Kalénine et al. (2013) required the processing of action end-goal, since the participants had to discriminate two actions executed with the same body kinematics to obtain different outcomes; performance in this task resulted not to be associated to any specific lesion damage, albeit a marginally significant association was noted with damage to the inferior frontal cortex (Kalénine et al., 2013). Thus, the fourth study group suggests that understanding the immediate and end-goal of observed actions may involve the inferior frontal cortex. This is in keeping with two recent TMS studies showing that stimulation of the inferior frontal cortex affects the ability to match the immediate-goal (Cattaneo et al., 2010) or the end-goal (Jacquet and Avenanti, 2013) of two actions depicted in a video and in a picture (independently of the effector used to grasp/pull a ball as in the study of Cattaneo and colleagues; or independently of the type of grip being used to achieve the end-goal of a sequence of actions as in the study of Jacquet and Avenanti, 2013). No effect was obtained after stimulation of the anterior intraparietal cortex (Jacquet and Avenanti, 2013), suggesting that processing action end-goals at an abstract level (i.e., independent of action means) relies more on the frontal node of the AON. Thus, these brain lesion and brain stimulation findings converge with neuroimaging studies of action execution (Johnson-Frey et al., 2005) and observation (Grafton and Hamilton, 2007; Bach et al., 2010) and provide causative evidence for a partial division of labor between the parietal and frontal nodes of the AON: while the inferior parietal cortex may be more involved in processing the specific way an observed transitive action is performed, i.e., the action’s means of goal-oriented actions, the inferior frontal cortex appears also involved in coding action outcome and goal at a more abstract level and may use such abstract information to complete missing and ambiguous perceptual information about ongoing actions.

## CONCLUSION

In sum, our ALE meta-analysis of studies using lesion-symptom mapping methods to describe the causal relation between brain lesions and action perception and understanding deficits identified three regions of the AON, namely the inferior frontal cortex, the inferior parietal cortex and the MTC/STC, whose damage was consistently associated with poor performance in action perception and understanding tasks that required to extrapolate biological motion from point-light displays, to match the kinematics of transitive and intransitve actions and to infer their end-goal. Interestingly, these areas correspond to the three nodes of the AON that are strongly activated in response to visual action perception in neuroimaging research (Caspers et al., 2010; Molenberghs et al., 2012a) and that have been targeted in previous brain stimulation studies (see Avenanti et al., 2013b for a review). Thus, brain lesion mapping provides converging evidence that premotor, parietal and temporal regions play crucial and possibly complimentary roles in perceptual and cognitive action-related processes.

Here we attempted to classify the different studies on the basis of the tasks used to probe action perception and comprehension and have highlighted the importance of differentiating between transitive and intransitive actions and between processing of different types of action-specific information (i.e., action means vs. action goal). However, the limited number of studies available in literature prevented us to draw strong conclusions from this classification and more empirical studies are needed in order to increase the robustness of the meta-analytic approach and to perform more specific task analyses. Furthermore, other action dimensions should be taken into account in the study of the neural bases of action perception disorders. In particular, neuroimaging studies have shown that observing upper and lower limbs and mouth actions activates different sectors of the premotor and parietal cortices in accordance with the somatotopic organization of movement execution (Buccino et al., 2001; Grosbras et al., 2012) and a recent brain stimulation study also supports this organization, with lip and hand motor representations in the premotor cortex being critically involved in processing observed mouth and hand actions, respectively (Michael et al., 2014). Most studies considered in this meta-analysis used only upper-limb movements, thus making it difficult to evaluate the possible role of somatotopy in the precise extent and localization of the neural underpinnings of action recognition. The two studies using point-light displays (Saygin, 2007; Han et al., 2013) showed whole body movements, which involved the displacement of both upper and lower limbs, thus preventing any consideration about somatotopic organization. Moro et al. (2008) used static images that implied actions of lower or upper limbs, but no dissociation between deficits in recognizing upper or lower limbs was noticed. Finally, Pazzaglia et al. (2008b) tested patients with buccofacial or limb apraxia and found a specific functional correspondence between deficits in imitating and matching mouth or upper limb actions. Lesion mapping analysis further confirmed that while insula damage was common to deficits in matching mouth and limb actions, deficits in matching limb actions were associated to damage of the inferior frontal cortex and inferior parietal cortex; conversely deficits in matching mouth actions were associated to damage of only inferior frontal cortex (Pazzaglia et al., 2008b). This last result seems in keeping with the involvement of inferior parietal cortex in coding hand–object interactions in transitive actions (Buccino et al., 2001).

A further important factor that should be taken into account when making inferences about the neural substrate of action perception is whether the action has or does not have a known functional, symbolic, or communicative meaning for the observer. Neuropsychological (e.g., Tessari et al., 2007) and neuroimaging (Peigneux et al., 2004; Rumiati et al., 2005) studies have shown dissociation between the neural correlates of imitating meaningful and meaningless actions. In a similar vein, using positron emission tomography (PET), Decety et al. (1997) found that observing meaningful vs. meaningless actions, with the instructions to either imitate or recognize them, activated partially dissociated neural networks within and outside the classical AON. Crucially, with the exception of Moro et al. (2008), who used both meaningful and meaningless actions, all studies entered in this meta-analysis presented only meaningful actions which were familiar to the observers. This limits the implications of the results to the perception and understanding of meaningful actions; different areas may be required when observers perceive new and meaningless movements of other individuals.

Although we found that damage to all three clusters in the inferior frontal and parietal cortex and MTC/STC caused action perception deficits, the relative involvement of these areas in action perception might be related to the amount of motor simulation required to complete ambiguous perceptual information (Avenanti et al., 2013a), to the domain-specificity of the observer’s motor expertise (Calvo-Merino et al., 2006; Aglioti et al., 2008; Fourkas et al., 2008; Abreu et al., 2012; Tomeo et al., 2013; Candidi et al., 2014; Makris and Urgesi, 2014) and to the level of action knowledge that needs to be inferred about others’ behavior. Notably, much less evidence has been provided by brain lesion studies on the ability to infer the final intention of the observers and to decide, for example, whether other are deceiving or providing genuine information on their ultimate aims. Although neuroimaging (Grezes et al., 2004; Iacoboni et al., 2005) and brain stimulation studies (Tidoni et al., 2013) suggest that the inferior frontal cortex may play a major role in these high-level action tasks, future studies are needed in order to provide converging causative evidence on how brain lesions may affect the ability to understand the ultimate intentions of others.

## Conflict of Interest Statement

The authors declare that the research was conducted in the absence of any commercial or financial relationships that could be construed as a potential conflict of interest.
